# The Specific Immune Response after Vaccination against Neonatal Calf Diarrhoea Differs between Apparent Similar Vaccines in a Case Study

**DOI:** 10.3390/ani11051238

**Published:** 2021-04-25

**Authors:** Román Gonzalez, Laura Elvira, Carlos Carbonell, Geert Vertenten, Lorenzo Fraile

**Affiliations:** 1 Practitioner SAT Prolesa, 27614 Galicia, Spain; prolesasat@yahoo.es; 2 Ruminant Technical Team, MSD Animal Health Spain, 37188 Salamanca, Spain; carlos.carbonell@merck.com; 3 MSD Animal Health, Wim de Körverstraat 35, 5831 AN Boxmeer, The Netherlands; geert.vertenten@merck.com; 4 Department of Animal Science, University of Lleida—Agrotecnio Center, 25198 Lleida, Spain; lorenzo.fraile@ca.udl.cat

**Keywords:** immune response, cow, neonatal diarrhoea, vaccination, passive immunity

## Abstract

**Simple Summary:**

Neonatal calf diarrhoea (NCD) is a complex, multifactorial disease involving pathogens but also environmental, nutritional and management factors. Enterotoxigenic *Escherichia coli* (ETEC), rotavirus (BoRV) and coronavirus (BoCV) are the main enteric pathogens involved. NCD is a major health challenge that impacts on farm profitability, calf welfare and antimicrobial usage. Thus, a key part of prevention is optimisation of preventive programs for NCD such as vaccinating cows to increase passive immunity transfer for their offspring during the neonatal period. In this study, it was demonstrated that dam vaccination against NCD is an excellent tool to increase specific immunity; how different vaccines differ in their specific immune response and, consequently, in the amount of specific passive transfer of antibodies to their calves. One of the most relevant findings is the importance of inducing a specific immune response for the three NCD pathogens.

**Abstract:**

Neonatal calf diarrhoea (NCD) is a major health challenge with a negative impact on farm profitability, calf welfare and antimicrobial use. Neonatal calves are particularly sensitive to enteric infections. Thus, a key point for prevention is minimising infectious pressure and maximising specific immune responses. An amount of 120 dams not previously vaccinated against NCD were randomly allocated to one of three study groups: negative control versus two vaccinated groups (A and B). In the control group, the average level of antibodies was significantly low for both BoCV and ETEC (15.6 and 13.9% in the colostrum samples, respectively), demonstrating the importance of dam vaccination. Indeed, the level of specific immunity was significantly increased for BoCV and ETEC with dam vaccination using both one-shot vaccines versus the control group. Moreover, the statistical analysis revealed a significantly higher level of antibodies for BoCV and ETEC in colostrum samples in vaccine A versus vaccine B and the control group. In accordance, the calf serum demonstrated a significantly higher level and greater homogeneity of antibodies against BoCV and ETEC in the Vaccine A group versus other experimental groups (*p* < 0.05). In conclusion, this study demonstrated a different specific immune response for the pathogens depending on the vaccine used to control NCD in cows.

## 1. Introduction

Youngstock rearing has an important role for future dairy cow performance. Indeed, colostrum management, health, average daily weight gain or calf nutrition have been directly related with future adult dairy cow production [1,2,3]. Moreover, maximising calf health could be an important aspect to allow heifers to express their full productive potential [4], as well as for their future survivability and productivity (days in milk, DIM) in the herd [5].

The preweaning period is the most disease-susceptible phase in the heifer-rearing period. Neonatal calf diarrhoea (NCD) is one of the major health challenges in dairy farms [6], being responsible for more than 50% of the morbidity in preweaned dairy calves, especially during the first three weeks of life [7,8], and the main cause of death in the first 30 days of life [8]. In addition to the cost of mortality and treatment of sick calves, the economic consequences of calf-hood diseases may include reduced growth performance, as well as increased age at first calving and reduced milk yields [9,10,11]. Consequently, NCD has a negative impact on cattle operation profitability [12] and creates a problem in terms of animal welfare, farmer wellbeing, and prudent use of antimicrobials [8,12].

NCD is a complex, multifactorial disease involving pathogens but also environmental, nutritional and management factors [13]. Worldwide, enterotoxigenic *Escherichia coli* (ETEC), rotavirus (BoRV), coronavirus (BoCV), and *Cryptosporidium parvum* are the pathogens most frequently involved in NCD, but other pathogens, such as *Salmonella* spp. or *Clostridium* spp., can also cause diarrhoea in calves [14,15,16,17,18]. The pathogens may act in isolation or more frequently in mixed infections, increasing the clinical severity and the age range of the affected animals [16,17]. Clinical disease is the result of an unfavourable balance between the resistance of the calf and infectious pressure [19]. Therefore, one key part of NCD prevention consists of maximising the calf immune response [20] to achieve a high level of balanced protection against the main enteric pathogens involved.

Colostrum is crucial for providing the neonates’ immunological protection during the first weeks of life [21], being one of the most important management factors in youngstock health and survivability [22]. Related to NCD, dam vaccination in the last months prior to calving, using appropriate adjuvant and antigen dose, has been long recognised as a reliable way to trigger the immune response, leading to the presence of protective immunoglobulins against the three pathogens (ETEC, BoRV and BoCV) in the dam’s serum, colostrum and transition milk [23,24]. Moreover, this increase in protection is transferred to the calf via the passively acquired immunoglobulins after ingestion of the vaccinated dam’s colostrum [23]. In both dairy and beef, it is described that having a high antibody titre against these three pathogens in the serum of calves is well correlated with the protection of these animals [25,26,27,28,29]. Therefore, different vaccines have been designed to increase the magnitude and duration of specific antibody levels [24].

In the last 25 years, two commercial vaccines have been licenced for a single dose administration in the last trimester of gestation, reducing handling and its associated risks, and thus having a positive impact on animal welfare [23,30]. Veterinary practitioners ask regularly for the differences between these vaccines as they have a similar product profile. This can only be evaluated if the vaccines are used in the same conditions. Both vaccines have been previously compared in different circumstances [31], but never in herds that have not been previously vaccinated against NCD. As this first vaccination is very important to establish a good immunological foundation, the best comparison should be made in herds not previously vaccinated against NCD. Consequently, the main aim of this blinded and randomised study was to assess the specific immune response induced for the three pathogens: BoRV, BoCV and ETEC after injection of one of two different one-dose commercially available NCD vaccines.

## 2. Materials and Methods

### 2.1. Ethical Statement

All experimental procedures were approved by the Ethics Committee for Animal Experimentation of the University of Lleida and performed in accordance with authorisation 7700 issued by the Catalonian Department of Agriculture, Livestock, Fisheries and Food (Section of Biodiversity and Hunting).

### 2.2. Study Design

This was a randomised, blinded, reference-controlled study with three study groups: two different vaccine groups (A and B) and one negative control group (non-vaccinated). The immune response in the dams and the passive immunisation of the calves was compared by measurement of specific antibody levels (assessed by commercial ELISA) in the serum of cows and calves, the colostrum (first milking after calving) and transition milk (milk from 8th milking) of the dams.

The study was conducted between September 2018 and January 2019. A single 400-cow milking Holstein–Friesian dairy farm located in the north of Spain, with no history of vaccination against major NCD antigens (BoRV, BoCV and ETEC) was enrolled in the study. A sample size calculation was carried out to ensure statistical power to detect differences between experimental groups based on biologically relevant differences in vaccine response (BoRV, BoCV and ETEC in the serum, colostrum and transition milk of dams). Forty dams per group were deemed adequate to detect 20% difference in antibody titre (60 ± 19% versus 72 ± 19%) between groups with a confidence level of 95% and a power of 80%. One hundred and twenty pregnant animals were enrolled in the trial, 12 to 5 weeks before the expected date of calving. The cows were randomly allocated to one of three study groups using a computer-generated randomisation code. The person responsible for vaccinating the cows was different from the practitioner responsible for the sampling and follow up of the animals. Randomisation was accomplished in advance according to a randomised complete block design based on their parity and the interval between inclusion (day 0 of vaccination) and the expected date of calving.

All vaccinated animals received one dose of an inactivated vaccine containing antigens of BoRV, BoCV and ETEC applied by intramuscular injection. Animals in the Vaccine A Group were administered with 2 mL of Bovilis^®^ Rotavec^®^ Corona (MSD-Animal Health, Boxmeer, The Netherlands) containing BoRV strain UK Compton, serotype G6 [P5]; BoCV strain Mebus; and the ETEC K99 adhesin antigen. This vaccine contains aluminium hydroxide and Montanide ISA 70 VG as adjuvants, while animals in the Vaccine B Group were administered 3 mL of Bovisan®/Bovigen® Scour (Virbac, Carros, Provence-Alpes-Cote D’Azur, France) containing BoRV strain TM-91, serotype G6 [P1]; BoCV strain C-197; and ETEC strain EC/17 expressing K99 adhesin. The adjuvant used in this vaccine is a water-in-oil-in-water commercial preparation (Montanide ISA 206 VG, Seppic, Paris, France). Animals in the control group were managed as usual without vaccination.

### 2.3. Husbandry of Animals

The animals were housed at a conventional dairy farm in Spain in accordance with standard husbandry practices. The study animals were co-mingled and housed in the same accommodation as non-study animals. The cows were fed a total mixed ration for pregnant dairy cows during the complete dry period based on grass silage, concentrate and straw (55% DM, 14% Protein, 56% NDF and 0.7 UFL) freshly prepared by a local cooperative and distributed daily to the farm. The dry cows were housed in a free stall barn with low stocking density (1.8 cubicles and 1.12 headlock per dry cow) and were moved to the maternity pen two days before the expected date of calving or when the animals start to show signs of upcoming parturition, whichever occurred first.

After calving, to ensure a good passive transfer of immunity, the newborn calves were separated from the dam within 3 h of parturition and colostrum was collected within 6 h after calving. The calves were then fed around 2.5 L of single-source colostrum within 6 h after birth, followed by a second administration of the same amount of colostrum 6–12 h later. No differences in colostrum management occurred by sex or study group.

### 2.4. Collection of Samples

Blood samples were collected from the coccygeal vein of the dams before vaccination (Serum0), on the day of vaccination (D0), and 0–6 h after parturition (SerumPart). Calf blood samples were collected during the first five days of life from the jugular vein (SerumCalf). Blood was collected in labelled 10-mL tubes with a clot activator (Vacutainer™ Serum Tubes). After collection, blood samples were kept at 15–25 °C for a minimum of 30 min, refrigerated and then were centrifuged for 7 min at 3000 rpm (equivalent to 1000× *g* force) to collect the serum. Colostrum was collected in the milking parlour at the first milking, and transition milk was collected at the 8th milking (8thMilk) of the cows. In both cases, samples were collected into labelled and sterile 10 mL plastic tubes and frozen before being sent for analysis. All samples were frozen at −20 °C for further testing.

### 2.5. Test and Analysis

Antibody titres against BoRV, BoCV and ETEC were assessed by measuring the inhibition of optical density (percentage of inhibition, PI) by competitive enzyme-linked immunosorbent assay (ELISA), on serum and colostrum. The BIO K 126 Monoscreen Ab ELISA Bovine Rotavirus; BIO K 392 Monoscreen Ab ELISA Bovine Coronavirus and BIO K 295 Monoscreen Ab ELISA *E. coli* F5 (K99) (all from BIO-X Diagnostics, Belgium) were used for the indirect quantification of antibodies against BoRV, BoCV and ETEC F5 (K99), respectively. The tests were performed blinded in a GLP accredited laboratory (Center for Diagnostic Solutions, Intervet International, Boxmeer, The Netherlands). The different tests were performed according to manufacturer’s instructions for serum and colostrum samples. For serum a dilution 1:20 was used in all cases, while for colostrum a dilution 1/20 in BIO K126 1/1000 in BIO K392 and 1:2 in BIO K295 was used. For intermediate milk a dilution 1/20 in BIO K126 and BIO K392; and 1:2 in BIO K295 was used.

### 2.6. Statistical Analysis

All statistical analyses were carried out in a blinded way using SAS V.9.1.3 (SAS institute Inc., Cary, NC, USA) by one of the authors. For all analyses, the individual dam or calf was used as the experimental unit. The significance level was set at 0.05 with statistical tendencies reported when *p*-value < 0.10. Firstly, a baseline homogeneity analysis was performed for the parity and the calving–vaccination interval between the different groups (Vaccine A, B and control) to assess that there were no significant differences between them.

Two statistical analysis were carried out. In the first one, the explanatory variable was the vaccine applied (A, B or control) and the outcome variables in the cows were antibody titre for BoRV, BoCV and ETEC K99 before vaccination (Serum0), at parturition (SerumPart), colostrum and 8th milking (8thMilk) of the cows (transition milk). The outcome variables in the calves were antibody titre for the former pathogens during the first five days of life (SerumCalf). Shapiro Wilk’s and Levene tests were used to evaluate the normality of the distribution of the continuous variables and the homogeneity of variances, respectively. A mixed model was performed to test the association between the experimental groups (Vaccine A, B and control) with the outcome variables for cows and calves taking into account repeated measures structure and the effect of the cow. The qualitative evaluation of this model was carried out with Q-Q plots and normality of residuals.

In the second statistical analysis, univariable models for BoRV, BoCV and ETEC K99 antibody response in cows were created using the following variables: physiological status (before vaccination-Serum0, delivery-Serumpart and 8th milking-8thMilk) and vaccination group (A, B or control) with cow as a random effect. If the significance of the variable was <0.2, it was added to the multivariable model in a forward selection process where the antibody response wasthe dependent linear variable and the explanatory variables were the physiological status (time points as categorical variable, with delivery-Serumpart as reference) and treatment group (A, B, control, with vaccine A as reference). Moreover, two-way interactions were assessed between the effects of the variables retained in the multivariable model. In the final model, variables and interactions were considered significant with a *p*-value <0.05.

## 3. Results

### 3.1. Cow Results

#### 3.1.1. Basal Homogeneity in the Groups—Including Results at Day 0

A total of 104 cows calved successfully and were included in the trial: 35 in vaccine Group A, 35 in vaccine Group B and 34 in the non-vaccinated group. Sixteen cows were excluded from the study: five cows with problems at calving or abortion, three cows due to inclusion errors and eight cows that calved before expected date for trial follow up. The cows were homogenously distributed (*p* > 0.05) among groups by parity (2.10; 2.48 and 2.5 for groups A, B and control, respectively) and interval of days (*p* > 0.05) between vaccination and calving (52.3 ± 16.3; 57.0 ± 16.5 and 57.5 ± 18.7 for groups A, B and control, respectively). Colostrum management was not significantly different (*p* > 0.05) between the different study groups for time from calving to colostrum milking (2.5; 2.5 and 2.1 h for group A, B and control, respectively), amount of colostrum intake during first feeding (2.53; 2.54 and 2.53 L for group A, B and control, respectively) and time from birth to colostrum intake (3.42; 3.39 and 3.52 h for group A, B and control, respectively).

In total 108 calves were born but five calves were lost within calving and the first 24 h of life (all of them belonged to the Control Group). Four pairs of twins were born (one in Vaccine A, one in Vaccine B and two in the Control Group).

#### 3.1.2. Immunological Responses against BoRV, BoCV and ETEC K99 in Cows

Immunological results in cows’ sera, colostrum and intermediate milk and calves’ sera, are presented in Table 1, as a percentage of inhibition ELISA (PI). The residuals of the mixed model showed normality reinforcing the robustness of the analysis.

**Figure 1:**
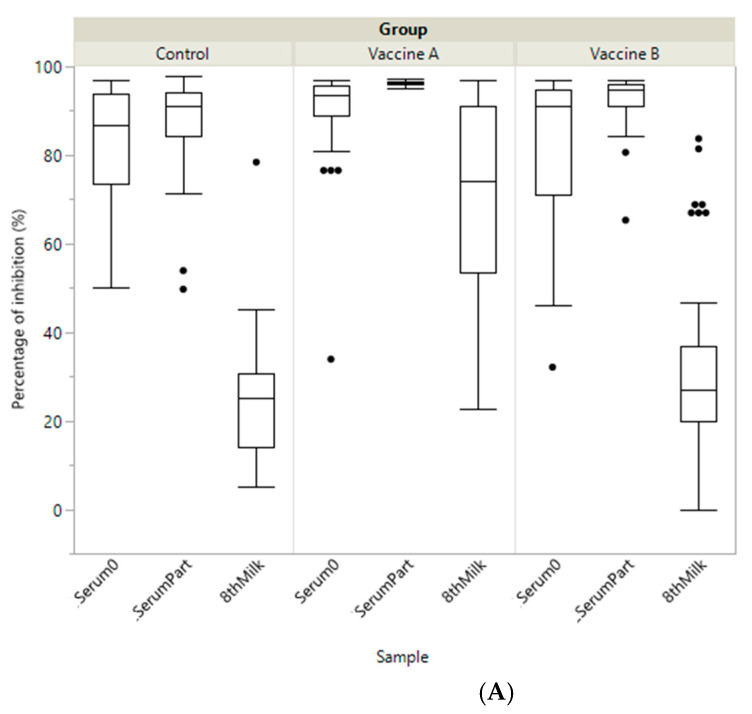

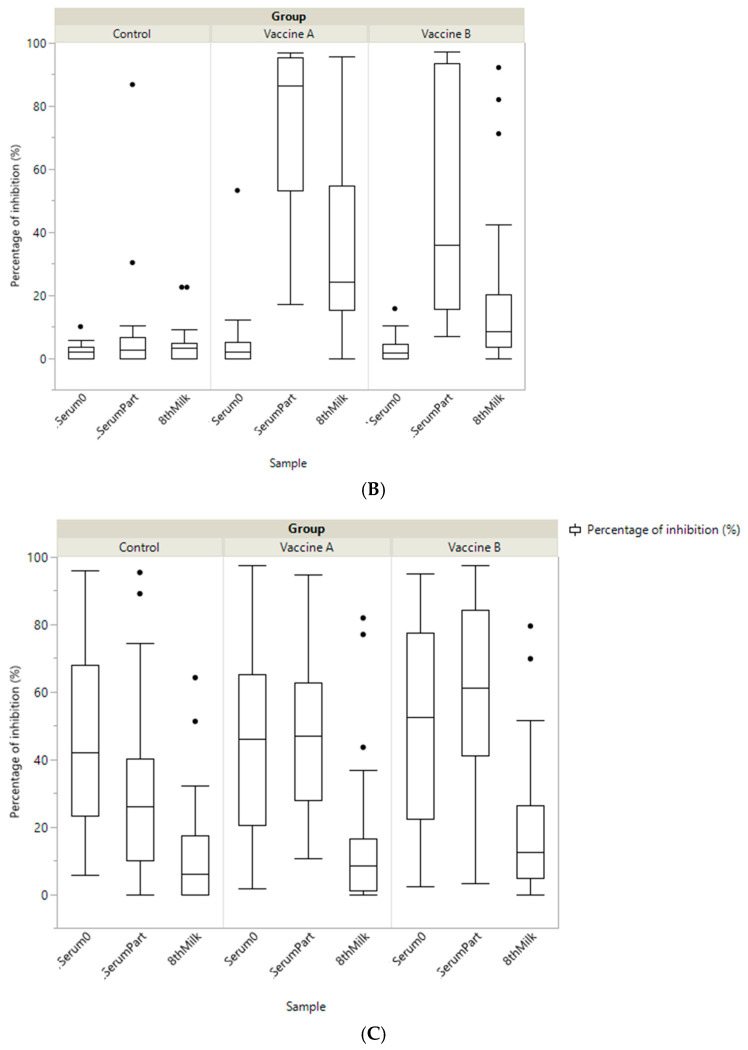
Boxplot showing specific immunity represented as percentage of inhibition (PI) of antibody titre including the different samples (Serum0, SerumPart and 8thMilk) by study group (Vaccine A, Vaccine B and Control) against: (**A**) coronavirus (BoCV); (**B**) enterotoxigenic *E. coli* (ETEC F5 (K99)) and (**C**) Rotavirus (BoRV).

**Figure 2:**
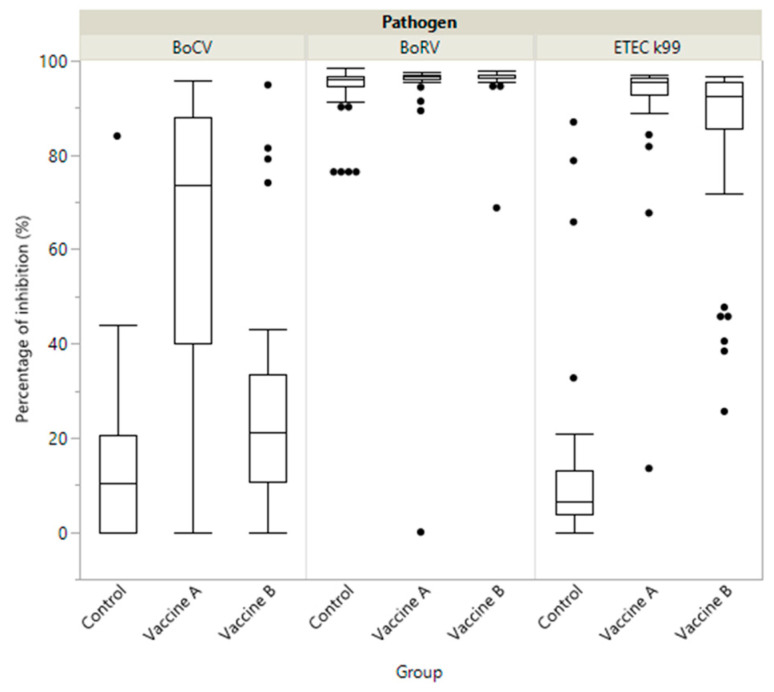
Boxplot showing specific immunity represented as a percentage of inhibition (PI) of antibody titre in colostrum by study group (Vaccine A, Vaccine B and Control) against: coronavirus (BoCV); enterotoxigenic *E. coli* (*ETEC K99*) and Rotavirus (BoRV).

Before vaccination, average PIs measured from dams’ sera (Serum0) were intermediate–high, intermediate but heterogeneous and very low for BoCV, BoRV and ETEC, respectively. At calving PI was significantly higher for vaccine A than for vaccine B and the control in dams’ serum (SerumPart), colostrum and transition milk (8thMilk) for both BoCV and ETEC (Table 1 and Figure 1). Moreover, in the control group the level of antibodies in colostrum samples was very low for BoCV and ETEC (15.6 and 13.9%, respectively), but not for BoRV (93.4%). For BoRV, PI was significantly higher between both vaccine groups and the control group for colostrum, and for vaccine B compared to vaccine A and the control group at calving (SerumPart). Finally, BoRV titres decreased in the control group from Serum0 to SerumPart and increased for both vaccine groups between these sampling times (Table 1 and Figure 1 and Figure 2).

#### 3.1.3. Multivariable Analysis for BoRV, BoCV and ETEC Antibody Titre in Cows

The BoRV, BoCV and ETEC antibody titres in cows were significantly affected by physiological status (before vaccination—Serum0, delivery—SerumPart and 8th milking—8thMilk) and vaccination group (A, B or control) in the univariable statistical analysis. BoCV and ETEC antibody titres in cows were significantly associated with physiological status (before vaccination-Serum0, delivery-SerumPart and 8th milking-8thMilk) and vaccination group (A, B or control) and its interaction in the multivariable analysis across the study period (Table 2). The goodness of fit was 0.72 and 0.77 for BoCV and ETEC, respectively. Moreover, significant differences in the vaccination variable were observed (*p* < 0.05) between the vaccine A group versus vaccine B and control groups for both pathogens (BoCV and ETEC), with the vaccine A group being superior to the other two groups across the study period (vaccine B and control).

In the case of BoRV antibody titres in cows were also significantly associated with physiological status (before vaccination-Serum0, delivery-SerumPart and 8th milking-8th Milk) and vaccination group (A, B or control) and its interaction in the multivariable analysis (Table 2). No significant differences were found between both vaccine groups for BoRV. The only significant difference observed (*p* < 0.05) was between the vaccine B and control groups for BoRV. Finally, the goodness of fit was 0.69 for BoRV.

### 3.2. Calf Results

#### Serology against BoRV, BoCV and ETEC K99 in Calves

**Figure 3:**
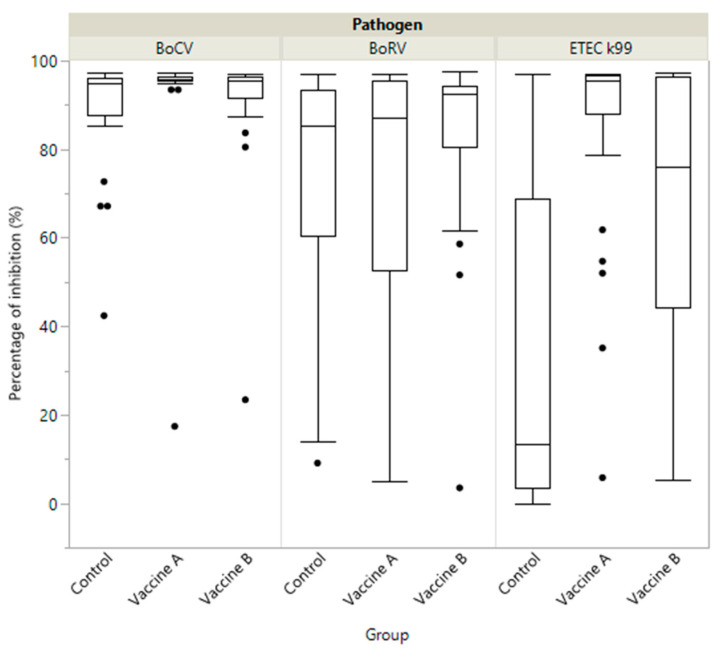
Boxplot showing specific immunity represented as percentage of inhibition (PI) of antibody in calves by study group (Vaccine A, Vaccine B and Control) against coronavirus (BoCV), enterotoxigenic *E. coli* (ETEC K99) and Rotavirus (BoRV).

As in the colostrum, the serum of calves from the Vaccine A group had significantly higher levels of antibodies against BoCV and ETEC compared to the Vaccine B and control groups (*p* < 0.05), with no significant differences for BoRV (*p* > 0.05) (Table 1). As shown in the boxplot diagrams, the Vaccine A group showed not only a higher median in the antibody level, but also a greater homogeneity in the antibody response for BoCV and ETEC (Figure 3).

## 4. Discussion

Colostrum is essential for the neonatal calf providing an important source of nutrients, non-specific immune factors, and specific immunoglobulin protection against a variety of pathogens. A higher level of maternal calf IgG has been associated with lower calf morbidity [8,32]. However, some doubts exist regarding the relationship between the failure of passive transfer (FPT) and the probability of diarrhoea with contradicting results in different studies [11,33,34,35]. This could be explained by the complex nature of this multifactorial disease [13,36] and the cut-off used to identify FPT calves, which is still under discussion [22,37].

However, it is widely accepted that the increase in specific maternal antibodies against BoRV, BoCV, and ETEC through colostrum and transition milk, reduces the risk of diarrhoea, duration of diarrhoea, and shedding of pathogens [25,29,38,39]; as a result of local protection from specific antibodies in the intestinal lumen, complementary with antibodies transferred back from the serum to the intestine of neonatal calves [27,40,41]. Dam vaccination against BoRV, BoCV, and ETEC in the last trimester of gestation is the most practical and effective tool to increase specific antibody levels [24,42]. Indeed, a higher antibody titre against these three pathogens in the serum of calves is highly correlated with the protection of these animals [25,26,27,29].

In this study, the immune response was assessed by the percentage of inhibition (PI, reflecting antibody concentrations) in competitive BoRV, BoCV and ETEC F5 (K99) ELISA’s. This method was chosen as it is one of the most commonly used and accurate techniques to measure antibody concentration as well as utilised in many previous studies [30]; allowing comparison of the results. For those competitive ELISA’s the dilutions were used as recommended by the manufacturer.

The antibody levels in the cow’s serum prior to vaccination were intermediate–high (≥70%) for BoCV, intermediate (40–70%) but heterogeneous for BoRV and very low (≤40%) for ETEC. This is in accordance with previous studies, which showed that BoRV and BoCV subclinical infections are quite common in dairy cows and are considered as possible sources of infection to the neonate [42]. This is also in accordance with previous studies where a percentage of animals presented high serum antibody titres against BoRV and BoCV prior to vaccination [24], presumably due to previous exposure to these pathogens. It may be that the intermediate and high antibody levels prior vaccination favour the vaccination response as this vaccination will rather boost then primo-immunize the acquired immunity from the endogenous pathogens. On the other hand, the low level of ETEC is also in accordance with previous studies showing a low level of natural antibodies to K99 antigen in adult cows, resulting in negligible specific antibody titres in normal colostrum, leaving the newborn fully susceptible [23,43].

The majority of the maternal IgG present in the colostrum seems to be transported from the serum into colostrum [44,45]. Therefore, the level of antibodies in the colostrum and transition milk may be related with those in the dam’s serum [46]. In the first statistical analysis, the titre of antibodies in the dam serum at calving was significantly higher in vaccine A than in the other two groups for BoCV and ETEC, while vaccine B was found to be significantly higher for BoRV. However, these differences in the immune response were only maintained at colostrum and transition milk level for BoCV and ETEC; and not for BoRV. Moreover, the antibody titre for BoCV and ETEC in cows is consistently increased across the study for vaccine A versus Vaccine B and the control group using a multivariable analysis, whereas the antibody titre for BoRV in cows was only consistently increased across the study period for vaccine B versus the control group. Thus, similar results were obtained using two different statistical analyses reinforcing the results obtained.

The titre of antibodies induced by dam vaccination at colostrum and transition milk level is very important for the protection induced in the calf by passive transfer and for the local lactogenic immunity [20]. At colostrum and transition milk level, significant differences were observed (*p* < 0.05) between the vaccine A group versus the B and control groups for both pathogens (BoCV and ETEC), the vaccine A group being superior to the other two groups. For BoRV the only significant difference observed (*p* < 0.05) was between the vaccine B and control groups. However, the control group also had a high PI level for BoRV in colostrum. This could be due to a previous field virus circulation in the dams, as previously described for BoCV and BoRV [42]. In contrast, other studies [30,42] described low levels of BoRV antibodies in the colostrum of non-vaccinated dams. Additionally, some studies have shown a significant increase in BoRV antibodies in the colostrum of cows vaccinated with both vaccines compared with the non-vaccinated dams [23,24,30].

Vaccine A induced the most balanced specific antibody response for the three studied pathogens. This immune response is in accordance with a previous comparative study, where dairy dams vaccinated with Vaccine A presented a significantly higher antibody immune response for the three antigens: BoRV, BoCV and ETEC, both in the colostrum and transition milk compared with another two-dose vaccine [24].

Finally, as in the colostrum, the serum of calves in Vaccine A group had significantly higher levels of antibodies against BoCV and ETEC (*p* < 0.05), with no significant differences against BoRV (see Table 1; *p* > 0.05). The level of antibodies for ETEC in calf’s serum was statistically superior in Vaccine A (86.7 versus 68.1% for Vaccine A and B, respectively). An interesting point to highlight is the agreement between the different results at dam level (serum at calving, colostrum and transition milk) and calf level (serum). In this study, passive transfer could not be assessed in individual calves to check colostrum intake, however this study was blinded and main factors affecting passive transfer, such as quantity of colostrum and time to colostrum milking and feeding, were standardized for all the calves. Thus, the colostrum administration should not introduce any bias into this study. Similar results were found in a previous study in the calf’s serum after the ingestion of colostrum from dams vaccinated with Vaccine B for BoRV and BoCV (85.1% and 74.0%, respectively) using the same quantification methodology [30]. Unfortunately, in this study the level for ETEC in the calf serum was not quantified and direct comparisons are therefore not possible [30].

It is likely that the design of vaccine A, including its antigen and adjuvants is related to the better immune response observed for BoCV and ETEC. Indeed, previous studies demonstrated the minimal increase in the levels of BoCV antibodies in either serum or milk after cow vaccination with different inactivated bovine coronavirus antigens. Vaccine A produced a significantly enhanced antibody response [47,48], which was explained by the authors as likely due to the level of coronavirus antigen in the respective vaccines and the use of a highly effective adjuvant [24,49]. Vaccine B also showed an increase in the level of antibodies for BoCV compared with the non-vaccinated dams [30]; however, in the present study, when both vaccines were compared, Vaccine A induced a significantly higher antibody response for BoCV both in colostrum and transition milk.

A further key point regarding NCD vaccines seems to be related to the ETEC protection. This pathogen is mainly involved in diarrhoea cases affecting calves for the first five days of life, but can be involved later in mixed infections [43]. Infected animals are the main reservoir for ETEC, and their faeces are the major source of environmental contamination. Indeed, less than 3 to 4% of dairy cows have natural antibodies to K99 antigen, as ETEC almost certainly does not affect adult animals [43]. For this important pathogen, vaccination with purified K99-containing vaccines has shown to be highly immunogenic [43]. Indeed, the Vaccine A group had a significantly higher level of antibodies in the colostrum and transition milk (91.3/34.9% versus 83.2/17.7% for Vaccine A and B, respectively). On the other hand, the PI for ETEC in the colostrum samples for Vaccine B group was very similar in a previous study with Vaccine B [43] (81.0% versus 83.2%, respectively). Moreover, in a previous study, vaccine B also showed a lower immune response to ETEC compared with the other pathogens included in the vaccine, suggesting that Vaccine B induced a less intense immune response to this pathogen [30]. This difference could be due to the type of ETEC antigen in both vaccines [30], as Vaccine A includes ETEC F5 (K99) adhesin antigen and other ETEC relevant proteins coming from the ETEC strain, while vaccine B includes inactivated ETEC strain EC/17 expressing adhesin F5 (K99). For parenteral administration, fimbrial-based vaccines are considered highly immunogenic for ETEC, as they are designed to increase the content of fimbrial antibody in the colostrum and transition milk. Fimbriae are considered very suitable protein antigens for inclusion in vaccination as they are located on the bacterial surface, readily accessible to antibodies and are required for a critical step in ETEC pathogenesis [43,50].

The titre of antibodies against ETEC and BoCV in the colostrum of the Control Group related to the non-vaccinated group was very low, (15.6 and 13.9%, respectively). However, a high level of antibodies against BoRV was present; perhaps due to a previous field virus circulation in the dams, as previously described for BoCV and BoRV [42], but not for ETEC [23,43]. This previous field virus circulation could be also responsible of the boosting effect of the vaccines observed. However, this situation could be different depending of the farm. Thus, other studies [30] have found low levels of BoRV antibodies in the colostrum of non-vaccinated dams (13.5%). Additionally, at calf level, the control group presented a significantly lower level of antibodies against ETEC and BoCV, with a larger difference at ETEC level (86.7, 68.1 and 33.9% for Vaccine A, B and control group, respectively). These results highlight the importance of including NCD vaccination in the farm preventive program, to achieve a high level of specific protection in the colostrum to be transferred to the calf.

It would have been ideal to check the efficacy of the different vaccines in the calves. This study paves the way to carry out additional studies focusing on efficacy in calves in order to evaluate at farm level the expected improvement of health outcomes after applying certain vaccines in cows to prevent NCD [51], the potential reduction in antibiotic use and the return of investment after implementing a neonatal diarrhoea prevention plan.

## 5. Conclusions

The results of this study demonstrated a low level of specific immunity against NCD in the colostrum and transition milk of non-vaccinated dams. This level of specific immunity is significantly increased with dam vaccination in the last trimester of pregnancy using both one-dose vaccines. However, one vaccine induced a significantly higher level of antibodies against ETEC and BoCV in the cows, and later in the calves after colostrum intake; without significant differences in BoRV antibody levels.

## Figures and Tables

**Table 1:** Antibody titre against BoRV, BoCV and ETEC K99 in serum, colostrum and 8th milk samples (values expressed as percentage of inhibition ELISA) in the dams of the different groups (Vaccine A, Vaccine B and Control). Additionally included are their calf’s serum values. Data are presented as median and confidence intervals (95%).

		BoCV			BoRV			ETEC	
	Vaccine A	Vaccine B	Control	Vaccine A	Vaccine B	Control	Vaccine A	Vaccine B	Control
Variable									
Serum0	89.2 ^a^	81.4 ^b^	82.6 ^b^	45.5 ^a^	50.6 ^a^	45.7 ^a^	4.2 ^a^	2.9 ^a^	2.1 ^a^
	(85.2–93.2)	(75.3–87.5)	(77.8–87.3)	(36.3–54.7)	(40.5–60.8)	(36.1–55.3)	(1.1–7.4)	(1.6–4.2)	(1.3–2.9)
SerumPart	96.3 ^a^	92.5 ^b^	86.9 ^c^	48.9 ^a^	61.6 ^b^	30.4 ^c^	72.8 ^a^	48.6 ^b^	6.6 ^c^
	(96.1–96.5)	(90.4–94.6)	(83.1–90.9)	(40.5–57.4)	(52.5–70.7)	(21.9–38.9)	(63.5–82.1)	(36.2–60.9)	(1.3–11.9)
Colostrum	64.1 ^a^	26.9 ^b^	15.6 ^c^	93.5 ^a^	95.8 ^a^	93.4 ^b^	91.3 ^a^	83.2 ^b^	13.9 ^c^
	(54.5–73.8)	(18.9–35.1)	(9.1–22.1)	(87.9–99.1)	(94.2–97.5)	(91.1–95.8)	(86.2–96.3)	(76.2–90.3)	(6.6–21.4)
8thmilk	69.0 ^a^	33.4 ^b^	24.7 ^b^	14.1 ^a^	18.6 ^a^	11.3 ^a^	34.9 ^a^	17.7 ^b^	3.9 ^c^
	(60.9–77.1)	(25.9–40.9)	(19.8–29.5)	(7.3–20.8)	(11.6–25.5)	(6.1–16.6)	(25.1–44.8)	(9.8–25.5)	(2.1–5.8)
SerumCalf	93.7 ^a^	91.7 ^b^	89.7 ^b^	76.7 ^a^	84.4 ^a^	73.9 ^a^	86.7 ^a^	68.1 ^b^	33.9 ^c^
	(89.3–98.2)	(87.6–95.9)	(85.3–94.1)	(68.2–85.2)	(78.3–90.6)	(64.5–83.4)	(79.8–93.5)	(57.7–78.3)	(20.8–47.2)

Different letters (a, b, c) in the same row and pathogen mean significant differences (*p* < 0.05) between experimental groups (Vaccine A, Vaccine B and control) using a mixed model.

**Table 2:** Multivariable analysis for BoCV, BoRV and ETEC antibody response in cows using physiological status (before vaccination-Serum0, delivery-SerumPart and 8th milking-8th Milk) and vaccination group (A, B or control) as explanatory variables with cow as a random effect. The reference value for physiological status and vaccination group in the multivariable analysis were delivery—SerumPart and vaccine A, respectively; CI = Confidence Interval 95%.

Outcome Variables	Variables	Coefficient (CI 95%)
BoCV antibody response		
	Physiological status	
	Before vaccination-Serum0	−7 (−14/0.10)
	8th milking—8th Milk	−27.3 (−34/−20)
	Vaccination group	
	Control	−9.3 (−16.4/−2.3)
	Vaccine B	−3.8 (−10.8/3.2)
Physiological status X vaccination group interaction	*p* = 0.009	
BoRV antibody response		
	Physiological status	
	Before vaccination—Serum0	−3.5 (−12.3/5.4)
	8th milking—8th Milk	−34.5 (−43.4/−25.6)
	Vaccination group	
	Control	−18.5 (−29.9/−7.1)
	Vaccine B	12.7 (1.3/24)
Physiological status X vaccination group interaction	*p* = 0.01	
ETEC antibody response		
	Physiological status	
	Before vaccination—Serum0	−68.6 (−76.5/−60.6)
	8th milking—8th Milk	−37.9 (−45.9/−29.9)
	Vaccination group	
	Control	−66.1 (−75.9/−56.5)
	Vaccine B	−24.2 (−33.8/−14.6)
Physiological status X vaccination group interaction	*p* < 0.001	

## Data Availability

The data presented in this study are available on request from the corresponding author. The data are not publicly available due to privacy restrictions.
